# Volatile Constituents from Different Parts of Three Lamiacea Herbs from Iran

**Published:** 2018

**Authors:** Shiva Masoudi

**Affiliations:** *Department of Chemistry, Central Tehran Branch, Islamic Azad University, Tehran, Iran.*

**Keywords:** Phlomis aucheri, Teucrium polium, Ajuga chamaecistus, Lamiacea, Essential oil, Hydrodistillation, Microwave extraction

## Abstract

The essential oils obtained by hydrodistillation from the stem, leaf and flower of *Phlomis aucheri *Boiss., which is endemic to Iran, stem, leaf and root of *Teucrium polium *L. and solvent free microwave extraction oil from leaf of *Ajuga chamaecistus *Ging. Subsp chamaecistus were analyzed by GC and GC/MS. Germacrene D (11.10%, 28.31% and 21. 06%) was the main constituent in the stem, leaf, and flower oils of *P. aucheri, *respectively. The other main component in the stem oil of the plant was (E) - anethole (24.58%) and in the flower oil was *β*- caryophyllene (15.93%). All three oils were rich in regard to sesquiterpenes. The main components in the stem, leaf and root of *T. polium *were *α*- muurolol (25.02%, 20.03% and 19.53%), *α*- cadinol (15.72%, 8.11% and 13.01%) and *β*-cayophyllene (10.86%, 10.11% and 10.64%) respectively. All three oils were rich in regard to sesquiterpenes. The major components in the leaf oil of *A.chamaecistus *were (z)-*β*-ocimene (12.11%) and germacrene D (10.11%). The oil of the plant was rich in regard to both monoterpens and sesquiter penes.

## Introduction

The genus *Phlomis *is comprised of about 100 species, 17 of them are described in the flora of Iran, among which 10 are endemic (1, 2). 

Some species of *Phlomis *are used in folk medicine as stimulants, tonics, diuretics and for the treatment of ulcers and haemorrhids (3-5). There are reports indicating various activities such as anti inflammatory, antinociceptive (4), antifibriel (6), antiallergic (7), antimalaria (8) and antimicrobial effects (9-11), for some species of this plant. 

Chemical studies on some *Phlomis *species have resulted different classes of glycosides containing diterpenoids (6), iridoids (11-13), phenyl propanoids (13), phenyl ethanoids (3, 5, 9), and flavonoids (14, 15). 

The genus *Teucrium *comprises about 340 species, 12 are described in the flora of Iran, among which three are endemic: *T.melissoides *Boiss et Hausskn ex Boiss., *T. macrum *Boiss et Hausskn and *T. persicum *Boiss. (1, 2). 

*Teucrium polium *is a perennial shrub, 20-50 cm high, distributed widely in the dry and stony hills and deserts of almost all Mediterranean countries, South western Asia, Europe and North Africa. 

In the traditional Iranian medicine, *T. polium *tea is used to treat oilments such as abdominal pain, indigestion, common colds, and urogenital diseases (16). 

Some biological and therapeutic effects have been reported for *T. polium *such as antioxidant (17), anti inflammatory (18), antinociceptive (19), anti pyretic (20), antimicrobial (17), hypolipidemic (21), hepatoprotective (22), antigastric ulcer (23), cytotoxic, and apoptotic effects (24). 

Several reports on the composition of volatile oils from *T. polium *are found in literature. In the oil from *T. polium *collected in Athens (Greece), *β*-caryophyllene(17.7%) was shown to be the major constituent accompanied by δ- cadinene (9.3%), caryophyllene oxide (5.9%) and *α*- cadinol (5.4%) (25). The oil of *T. polium *subsp. valentinum from Spain, was found to contain *α*-pinene (15.8%) and *β*-pinene (11.7%) as major constituents (26). *α*-Muurolene (8.7%), *α*- cadinol (5.9%) and δ-cadinene (5.1%) were found to be the major constituents of the essential oil of *T. polium *from northern region of Saudi Arabia (27). The dominant compounds in *T*. *polium *from Corsica were *α*- pinene (28.8 %), *β*- pinene (7.2%), and *p*-cymene (7.0%) (28). 

The genus *Ajuga *is represented in the flora of Iran by five species in which *Ajuga chamaecistus *has contained several endemic subspecies including *A. chamaecistus *ssp. chamaecistus (1, 2).

Several biological studies have been performed on many species of this genus which have confirmed their ethno pharmacological properties such as hypoglycemic (29), anti inflammatory (30), anabolic, analgestic, antiarthritis, antipyretic,hepatoprotective, antibacterial, antifungal, antioxidant, cardiotonic (31), and antimalarial (32) properties and also their application in the treatment of join diseases (33) . 

Many phytochemical studies on *Ajuga *species were performed which led to the isolation of phytoecdysteroids (34, 35), clerodane and neoclerodane di terpenoids (36, 37), iridoids (38) as well as phenylethyl glycosides (39). Previously, we studied the essential oil obtained by hydrodistillation from the aerial parts of *A. chamaecistus *Ging. ssp. chamaecistus, collected from Fasham, 35 km east of Tehran, which contained *β*- pinene (15.0%) and linalool (14.5%) as major constituents (40). 

Our study deals with the analysis of the oils from stems, leaves, and flowers of *Phlomi*s *aucheri*, from stems, leaves, and roots from *Teucrium polium *and also from leaves of *Ajuga chamaecistus *growing wild in Iran. 

## Experimental

*Plant materials*

The stems, leaves, and flowers of *Phlomis aucher*i, which is endemic to Iran, and stems, leaves and roots of *Teucrium polium*, were collected from Salehabad area, Province of Ilam, west of Iran, both in July 2013, during the flowering stage. The leaves of *Ajuga chamaecistus *were collected from Mehran, Province of Ilam, in July 2013. Voucher specimens have been deposited at the Herbarium of the Reasearch Instituents of Forests and Rangelands (TARI), Tehran, Iran. 

**Table 1:** Comparative percentage composition of the stem, leaf and flower oils of *Phlomis aucheri*

No.	Compounds^a^	RI^b^	Stem Oil (%)	Leaf Oil (%)	Flower Oil (%)
1	*α*-Thujene	928	0.12	-	-
2	*α*-Pinene	935	3.12	4.81	0.40
3	*β*-Pinene	978	0.13	0.19	0.12
4	Myrcene	991	0.15	-	-
5	*α*- Phellandrene	1003	0.18	-	-
6	Limonene	1031	0.62	0.57	0.33
7	Fenchone	1086	1.65	-	-
8	Linalool	1096	0.13	0.36	0.12
9	Nonanal	1098	0.12	0.19	0.14
10	Camphor	1143	0.47	0.21	-
11	Terpin-4-ol	1176	0.15	0.22	-
12	*α*-Terpineol	1189	-	0.23	-
13	methyl Chavicol	1195	0.47	-	-
14	endo-Fenchyl acetate	1220	0.23	-	-
15	exo-Fenchyl acetate	1232	0.61	-	-
16	*p*-Anisaldehyde	1252	0.30	-	-
17	(E)- Anethole	1283	24.58	-	-
18	Carvacrol	1298	-	2.29	0.47
19	Undecanal	1306	-	-	0.37
20	iso- dihydro Carveol acetate	1325	-	-	0.26
21	δ- Elemene	1339	0.32	3.68	3.93
22	Citronellyl acetate	1352	0.18	-	-
23	Neryl acetate	1365	4.58	-	-
24	*α-* Ylangene	1372	-	0.44	0.58
25	Butanoic acid- butyl ester	1373	-	0.43	-
26	*α*- Copaene	1374	0.76	1.40	1.63
27	*β*- Bourbonene	1384	0.55	2.69	1.05
28	*β*- Cubebene	1390	-	0.36	0.13
29	*β*- Elemene	1391	-	0.94	0.69
30	Tetradecane	1399	-	0.23	-
31	*α*- Gurjunene	1409	-	-	0.27
32	*β*-Caryophyllene	1418	5.58	4.96	15.93
33	*β*-Gurjunene	1432	0.15	1.12	0.81
34	γ-Elemene	1433	0.36	5.46	7.90
35	Aromadendrene	1439	-	0.43	-
36	*α*-Guaiene	1440	-	-	0.21
37	*α*- Himachalene	1447	-	-	0.39
38	*α*-Humulene	1454	0.85	2.01	3.46
39	(E)-*β*-Farnesene	1458	0.75	3.52	2.45
40	9-epi-(E)- Caryophyllene	1465	0.78	1.13	2.90
41	Germacrene D	1480	11.10	28.31	21.06
42	*β*-Selinene	1485	-	1.16	2.76
43	Viridiflorene	1493	2.38	-	-
44	Bicyclogermacrene	1494	6.30	8.86	7.63
45	Germacrene A	1501	0.63	-	-
46	γ-Cadinene	1511	-	-	0.18
47	7-epi-*α*- Selinene	1515	0.42	-	0.48
48	δ- Cadinene	1522	2.58	2.18	2.81
49	(z)-Nerolidol	1530	0.77	-	-
50	GermacreneB	1556	4.53	2.89	1.70
51	(E )- Nerolidol	1564	0.60	-	0.26
52	Spathulenol	1576	6.01	3.28	2.05
53	Caryophyllene oxide	1580	-	0.36	1.33
54	Globulol	1581	-	0.56	-
55	Viridiflorol	1590	0.52	0.51	0.57
56	Hexadecane	1600	-	0.23	-
57	Humulene epoxide II	1604	-	-	0.34
58	Iso spathuelenol	1637	-	0.41	0.35
59	epi-*α*- Muurolol	1639	-	-	0.57
60	*α*- Muurolol	1645	0.47	0.69	-
61	*β*- Eudesmol	1649	2.68	-	-
62	Selin-11-en-4-α- ol	1652	-	0.26	-
63	*α*- Cadinol	1653	1.01	0.91	0.74
64	*α*- Bisabolol	1680	0.72	-	-
65	( E,E )- Farnesol	1720	0.70	-	-
66	6,10,14- trimethyl 2-Pentadecanone	1845	1.15	2.05	1.28
67	Hexadecanoic acid	1973	0.35	-	-
68	Eicosane	2000	0.77	-	-
69	Tricosane	2300	-	0.51	0.88
					
	Monoterpene hydrocarbons		4.32	5.57	0.85
	Oxygenated monoterpenes		8.00	3.31	0.85
	Sesquiterpene hydrocarbons		38.04	71.54	78.95
	Oxygenated sesquiterpenes		13.48	6.98	6.21
	Other compounds		27.74	3.64	2.67
	Total		91.58	91.04	89.53

a Note: Compounds listed in order of elution from HP- 5 MS column;

b Retention indices to C_8_- C_24_ n-alkanes on HP- 5 MS column.

**Table 2:** Comparative percentage composition of the stem, leaf and root oils of *Teucrium polium*

**No.**		**Compounds** a		**RI** b		**Stem** **Oil(%)**	**Leaf** **Oil (%)**		**Flower** **Oil(%)**		
1		(E)-2- Hexenal		854		-	0.10		-		
2		α- Pinene		935		0.66	2.89		-		
3		Camphene		950		t	0.16		-		
4		*β*- Pinene		978		1.70	6.65		-		
5		Myrcene		991		0.37	1.51		-		
6		*p*- Cymene		1024		0.30	t		-		
7		Limonene		1031		0.56	3.12		-		
8		( E )-*β*- Ocimene		1050		0.22	0.32		-		
9		γ-Terpinene		1062		-	0.13		-		
10		Terpinolene		1086		t	0.18		-		
11		Linalool		1098		0.37	0.87		-		
12		1-Octen- 3 yl acetate		1110		-	0.60		-		
13		*trans* **- **Pinocarveol		1136		-	0.91		-		
14		*cis* **- **Verbenol		1140		t	0.69		-		
15		Pinocarvone		1160		-	0.47		-		
16		Borneol		1165		0.50	0.85		-		
17		Terpin-4- ol		1175		-	0.33		-		
18		Myrtenal		1193		-	1.20		-		
19		α- Fenchyl acetate		1220		0.26	-		-		
20		*cis*- Chrysanthenyl acetate		1262		0.71	1.01		-		
21		Bornyl acetate		1285		0.23	0.16		-		
22		2- methyl Naphthalene		1292		0.28	-		-		
23		δ- Elemene		1339		t	0.38		0.15		
24		α- Copaene		1374		0.44	0.35		0.18		
25		*β*- Caryophyllene		1418		10.86	10.11		10.64		
26		*trans* **-** *α*- Bergamotene		1434		0.42	1.07		-		
27		Geranyl acetone		1453		0.27	-		-		
28		α- Humulene		1454		2.25	3.08		0.75		
29		allo- Aromadendrene		1459		0.35	-		-		
30	Germacrene D		1480		0.84			2.77		1.23
31	Bicyclogermacrene		1494		0.98			2.02		-
32	*α*- Muurolene		1497		0.30			-		-
33	γ- Cadinene		1513		3.78			4.56		2.06
34	δ- Cadinene		1522		2.79			2.96		1.55
35	α- Calacorene		1542		0.27			-		-
36	(z)- Nerolidol		1534		-			7.13		-
37	Elemol		1549		5.53			3.17		3.50
38	Geranyl-n- butyrate		1560		0.58			-		-
39	(E)- Nerolidol		1564		2.51			2.26		2.28
40	Spathulenol		1576		-			2.37		-
41	*trans-*Sesquisabinene hydrate		1580		0.91			-		-
42	Caryophyllene oxide		1581		6.49			3.77		3.19
43	Dodecanoic acid		1589		1.26			-		-
44	Guaiol		1593		0.26			-		-
45	Tetradecanal		1611		0.62			-		-
46	*α*- Muurolol		1645		25.02			20.03		19.53
47	*β*- Eudesmol		1649		-			-		1.47
48	α- Cadinol		1653		15.72			8.11		13.01
49	Valerianol		1655		0.34			-		-
50	Khusinol		1675		-			0.54		-
51	(z, z)- Farnesol		1713		0.48			0.53		0.42
52	Cadina- 4,10(15)- dien-3- one		1740		0.35			0.37		-
53	Tetradecanoic acid		1771		0.67			-		-
54	6.10,14- trimethyl-2- Pentadecanone		1872		0.39			0.11		0.23
55	(z, z)- 9,12- Octadecadienoic acid		1953		0.83			-		1.06
56	(E)-9- Octadecenoic acid		1958		-			-		4.16
57	(z)- 9,17- Octadecadienal		1965		-			-		1.99
58	Hexadecanoic acid		1973		5.17			0.64		16.37
59	Eicosane		2000		-			-		4.93
					
	Monoterpene hydrocarbons				3.81			14.96		-
	Oxygenated monoterpenes				2.92			6.49		-
	Sesquiterpene hydrocarbons				23.28			27.30		16.56
	Oxygenated sesquiterpenes				57.61			48.28		43.40
	Other compounds				9.22			1.45		28.74
	Total				96.84			98.48		88.7

t= trace (< 0.1%)

a Note: Compounds listed in order of elution from HP- 5 MS column;

b Retention indices to C_8_- C_24_ n-alkanes on HP- 5 MS column;

**Table 3:** Percentage composition of the leaf oil of *Ajuga chamaecistus*

**No.**	**Compounds** a		**RI** b		**(%)**		
1	*α*- Thujene		928		0.55		
2	*α*- Pinene		935		4.42		
3	Camphene		950		0.23		
4	Sabinene		976		0.16		
5	*β*- Pinene		980		2.38		
6	6- methyl-5- Hepten-2- one		983		0.34		
7	1-Octen-3- ol		986		3.89		
8	Myrcene		991		0.77		
9	*α*- Phellandrene		1003		0.37		
10	*p*- Cymene		1024		1.05		
11	Limonene		1031		0.77		
12	(z)-*β*- Ocimene		1040		12.11		
13	(E)- *β*- Ocimene		1050		0.56		
14	γ-Terpinene		1062		1.58		
15	Octanol		1070		0.58		
16	Terpinolene		1088		0.46		
17	Linalool		1096		5.25		
18	Nonanal		1098		0.41		
19	all- Ocimene		1126		0.97		
20	*trans* **- **Pinocarveol		1137		0.17		
21	Camphor		1141		0.36		
22	*trans*-Verbenol		1142		0.62		
23	(E)-2- Nonenal		1157		0.18		
24	Lavandulol		1164		0.72		
25	Terpin-4- ol		1177		0.43		
26	*α*- Terpineol		1189		0.75		
27	Geraniol		1253		0.33		
28	Bornyl acetate		1285		6.08		
29		Lavandulyl acetate		1289		0.73
30		Carvacrol		1296		0.17
31		Eugenol		1356		0.49
32		α- Copaene		1376		0.68
33		Geranyl acetate		1381		0.64
34		*β*- Bourbonene		1383		0.78
35		*β*- Elemene		1391		0.40
36		(z)- Jasmane		1394		0.39
37		methyl Eugenol		1401		0.12
38		*β*- 1,7- di- epi Cedrene		1410		0.52
39		*trans*-*α*- Ambrinol		1412		1.31
40		*β*- Caryophyllene		1418		1.52
41		*β*- Gurjunene		1432		0.25
42		*trans* **-** α **- **Bergamotene		1436		2.39
43		α *-* Humulene		1454		0.62
44		( E )-*β*- Farnesene		1456		1.54
45		γ- Muurolene		1476		0.58
46		Germacrene D		1480		10.11
47		Bicyclogermacrene		1492		2.06
48		Cuparene		1502		0.19
49		*β*- Bisabolene		1507		0.71
50		γ- Cadinene		1513		0.50
51		δ- Cadinene		1524		1.15
52		Germacrene B		1556		0.21
53		(E)- Nerolidol		1564		0.56
**No.**		**Compounds** ^a^		**RI** ^b^		**(%)**
54		Spathulenol		1574		6.10
55		Caryophyllene oxide		1581		1.74
56		*β*- Oplopenone		1606		0.85
57		Cedr- 8(15)- en- 9- *α*- ol		1644		0.28
58		(z)- methyl Jasmonate		1647		0.75
59		(z)- Nerolidol acetate		1675		0.42
60		*α*- Bisabolol		1683		1.17
61		Tetradecanoic acid		1760		0.65
62		Hexadecanol		1819		0.40
63		( E, E)- Farnesyl acetate		1841		0.24
64		6,10,14- trimethyl -2- Pentadecanone		1843		0.63
65		6,10,14- trimethyl-5,9,13- Pentadecatrien-2- one		1915		0.17
66		Hexadecanoic acid		1970		3.06
67		(z, z , z) 9,12,15- Octadecatrienonic acid-methyl ester		2010		0.22
68		( E )-5- Octadecene		2083		0.81
			
		Monoterpene hydrocarbons				26.38
		Oxygenated monoterpenes				16.25
		Sesquiterpene hydrocarbons				24.21
		Oxygenated sesquiterpenes				11.36
		Other compounds				14.40
		Total				92.6

: a NoteCompounds listed in order of elution from HP- 5 MS colum;

b Retention indices to C_8_- C_24_ n-alkanes on HP- 5 MS column.

*Isolation of the essential oils*

*Hydrodistillation*

The stems (102.5 g), leaves (84.0 g), and flowers (82.0 g) of *P.aucher*i and also stems (110 g), leaves (80 g), and roots (65 g) of *T. polium *were separately subjected to hydrodistillation using a Clevenger-type apparatus for 3 h. After decanting and drying of the oils over anhydrous sodium sulfate the corresponding yellowish coloured oils were recovered [in the yield of 0.2%, 0.4%, 0.3%, 0.2%, 0.4% and 0.1% (w/w), respectively]. 

*Solvent- free microwave extraction*

Solvent-free microwave extraction (SFME) of the leaf of *A. chamaecistus *was performed in a Milestone ETHOM 1600 batch reactor, which is a multimode microwave reactor operating at 2455 MHz with a maximum delivered power of 1000 W, variable in 10 W increments. The dimensions of the PTFE coated cavity are 35×35×35 cm. During the experiment, time temperature, pressure, and power were controlled using the «easy-WAVE» Software package. Temperature was monitored with the aid of a shielded thermocouple (ATC- 300) inserted directly in to the sample container. 

In a typical SFME procedure, 250 g of dry leaves of *A. chamaecistus *were moistened prior to extraction by soaking in water for 1 h, then draining off the excess water. 

This step is essential to give the leaves the initial moisture. Moistened leaves were next placed in the reactor without any added solvent or water. The essential oil is collected, dried with anhydrous sodium sulphate and stored at 0 °C until used. 

*Gas chromatography analysis*

Gas chromatography analysis was performed on Schimadzu 15A gas chromatograph equipped with a split /splitless injector (25 °C) and a flame ionization detector (250 °C). Nitrogen was used as carrier gas () and the capillary column used was DB-5 (50 m × 0.2 mm, film thickness 0.32 . The column temperature was kept at 60 °C for 3 min and then heated to 220 °C with a 5 °C rate and kept constant at 220 °C for 5 min. Relative percentage amounts was calculated from peak area using a Schimadzu C- R4 A chromatopac without the use of correction factors. 

*Gas chromatography – mass spectrometry analysis*

Analysis was done using a Hewlett-Packard 5973 with a HP- 5 MS column (30 m × 0.25mm, film thickness 0.25). The column temperature was kept at 60 °C for 3 min and programmed to 220 °C at a rate of 5 °C and kept constant at 220 °C for 5 min. The flow rate of Helium as carrier gas with () MS was taken at 70 e V. 

The retention indices for all the components were determined according to the Van Den Dool method, using n- alkanes as standard (41, 42). 

The compounds were identified by (RI, DB5) with those reported in the literature and by comparison of their mass spectra with the Wiley library or with the published mass spectra (43). 

## Results and Discussion

The composition of the essential oils from stems, leaves, and flowers of *Phlomis aucheri*, stems, leaves and roots of *Teucrium polium *and leaves of *Ajuga chamaecistus*, are listed in Table 1, 2 and 3, respectively, in which the percentage and relative retention indices of components are given. 

As it is shown from Table 1, in *P. aucheri *we identified 46 compounds representing 91.58%, 40 constituents representing 91.04% and 40 components representing 89.53% of the stem, leaf and flower oils, respectively. The main component in three oils was germacrene D (11.10%, 28.31% and 21.06%), respectively. Other notable constituents were *β*- caryophyllene (5.58%, 4.96% and15.93%) and bicyclogermacrene (6.30%, 8.86% and 7.63%), respectively.

(E)- Anethole (24.58%) was the other main component in the stem oil of the plant and not detected in the leaf and flower oils. As can be seen from the above information, all three oils were rich in regard to sesquiterpenes (51.52%, 78.52% and 85.16%), respectively. The

monoterpene fraction was relatively small, representing only 12.32%, 8.88% and 1.70%, respectively. In the stem oil of *P. aucheri*, considerable percentage of non terpenoid compounds, compairing to other parts of the plant oils, were identified (27.74%). In an earlier study, Javidnia et al. analyzed the essential oil of the aerial parts of *P. aucheri *collected in Fars province. The oil was found to be rich in caryophllyene oxide (33.5%), *β*- caryophyllene (27.0%) and*β*-selinene (9.9%) (44). 

Only a few reports on the analysis of essential oils of *Phlomis *species have been published. 

Water distilled essential oils from aerial parts of *P. persica and P. olivieri*, which are endemic to Iran, have been the subject of our previous studies. The major components of both oils were germacrene D (38.2% and 26.4%) and bicyclogermacrene (16.3% and 12.7%), respectively. 

Both oils consisted mainly of sesquiterpene hydrocarbons (45). Also we reported the oil composition of *P. pungens*. The major constituents of the oil of the plant were germacrene D (24.5%), bicyclogermacrene (14.1%), α-pinene (13.5%) and (E) - *β*- farnesene (13.4%) (46). 

Camparison of the present results with those of our previous investigation of oils of the *Phlomis *genus showed that they are also dominated by sesquiterpenes. 

Germacrene D has been identified in other species of *Phlomis*, including *P. cancellata *(47), *P. bracteosa *(48), *P. armeniaca *(49), *P. chorassanica *(50), *P. herba-venti *(51), *P. bruguieri *(52, 53), *P. lanceolata*, *P. anisoonata *(53) and *P. linearis *(54). 

As it is shown from Table 2, 45 components representing 96.84%, 41 constituents representing 98.48% and 20 compounds representing 88.7% were identified in the oils of stem, leaf and root of *T. polium*, respectively. 

The main components in all three oils were *α-*muurolol (25.02%, 20.03% and19.53%), *α*-cadinol (15.72%, 8.11%, and 13.01%) and *β*-caryophyllene (10.86%, 10.11% and 10. 64%), respectively.

Other notable compounds were in stem oil; caryophyllene oxide (6.49%), elemol (5.53%) and hexadecanoic acid (5.17%), in leaf oil, (z) nerolidol (7.13%) and *β*- pinene (6.65%), in root oil; hexadecanoic acid (16.37%). 

According to these results, the composition of the stem, leaf and root oils of *T. polium *show significant similarity for the concentration of the main components. All three oils were rich in regard to sesquiterpenes (80.89%, 75.58% and 59.96%), respectively. 

The monoterpene fraction of the stem and leaf oils was relatively small, representing (6.73% and 21.45%) of the total oils, resepectively. In the root oil of the plant we could not find any trace of monoterpenes. In the root oil of *T.polium*, considerable percentage of non terpenoid compounds, compared to other parts of the plant oils, were identified (28.74%). 

Water distilled oil obtained from the aerial parts of *T. persicum*, which is endemic to Iran, have been the subject of our previous studies. epi-*α*-Cadinol (23.2%) and *α*-pinene (17.3%) were the main components among the thirty-one constituents characterized in the oil of *T. persicum *representing 95.9% of the total components detected (55). The oil of *T. gnaphalodes *was characterized by higher amounts or *β*-caryophyllene (12.1%), sabinene (8.8%) and *trans*- pinocarveol (7.8%) (53). 

As it is shown from the Table 3, in *A. chamaecistus *oil, 68 components, which representing about 92.6% of the total composition, were identified. The leaf oil of *A. chamaecistus *consists of 14 monoterpene hydrocarbons (26.38%), 12 oxygenated monoterpenes (16.25%), 17 sesquiterpene hy- drocarbons (24.21%), 8 oxygenated sesquiterpenes (11.36%), and 17 non terpenoid compounds (14.40%). The major components of this oil were (z)-*β*- ocimene (12.11%) and germacrene D (10.11%) followed by spathulenol (6.10%) and bornyl acetate (6.08%).

The leaf oil of *A. chamaecistus *was rich in regard to both monoterpenes (42.63%) and sesquiterpenes (35.57%). 

The qualitative and quantitative variation between our results and our previous reports (40) for the constituents of the oil from aerial parts may be attributed to the different environment conditions and different methods of extraction of the oils. 

The oils of the genus *Ajuga *have been the subject of only a few studies. The oil of *A. chamaecistus *subsp. tomentella contained thymol (34.5%) and exo- fenchol (15.6%) as the major components (56). 

The oil of *A. chamaecistus *subsp. scoparia was characterized by higher amounts of *p*-cymene (34.5%), *β*- pinene (18.0%), *α*- phellandrene (17.8%) and *α*- pinene (15.2%) were major constituents (57) . 

The major constituents of the aerial parts of *A. chamaepitys *ssp. chamaepitys were *β*-pinene (34.3%) and *α*-pinene (16.1%) (58). The dominant compounds in *A. bombycina *were *β*- pinene (28.2%) and *α*- pinene (18.5%) (59). 
